# Advance Statements for Black African and Caribbean people (AdStAC): protocol for an implementation study

**DOI:** 10.1186/s12888-023-04825-6

**Published:** 2023-05-17

**Authors:** Abigail Babatunde, Alex Ruck Keene, Alan Simpson, Steven Gilbert, Lucy Stephenson, Kia-Chong Chua, Gareth Owen, Adelabu Jonathan Simpson, Shubulade Smith, Claire Henderson

**Affiliations:** 1 Health Services and Population Research Department, David Goldberg Centre, King’s College London Institute of Psychiatry, Psychology and Neuroscience, De Crespigny Park, London, SE5 8AF UK; 2 Institute of Psychiatry, Psychology and Neuroscience, 39 Essex Chambers, London, UK; 3 Steve Gilbert Consulting, Blackthorn House, St Pauls Square, Birmingham, BC 1RL UK; 4 Department of Psychological Medicine, King’s College London Institute of Psychiatry, Psychology and Neuroscience, De Crespigny Park, London, SE5 8AF UK; 5 Maudsley Hospital, Denmark Hill, London, SE5 8AZ UK

**Keywords:** Advance statements, Advance choice documents, Mental Health Act, Advance directives, Black mental health, New Mental Health Act

## Abstract

**Background:**

The UK government committed to legislating for Advance Choice Documents/Advance Statements (ACD/AS) following their recommendation by the Independent Review of the MHA (2018). ACDs/AS are yet to be implemented in routine practice despite evidence and high demand; they are associated with improved therapeutic relationships and a reduction (25%, RR 0.75, CI 0.61–0.93) in compulsory psychiatric admission. Barriers to their implementation are well documented, ranging from low knowledge levels to logistical challenges in accessing the content during episodes of acute care. In the UK this is an issue for Black people, who experience detention rates disproportionately (over three times) higher than those of White British people and have poorer care experiences and outcomes. ACDs/AS allow for Black people to have their concerns heard by mental health professionals in a care system where they often feel their views are ignored. AdStAC aims to improve Black service users’ experiences in mental health services in South London by co-producing and testing an ACD/AS implementation resource with Black service users, mental health professionals and carers/supporters of Black service users.

**Methods/design:**

The study will take place in South London, England over three phases: 1) formative work through stakeholder workshops; 2) co-production of resources through a consensus development exercise and working groups; and 3) testing of the resources using quality improvement (QI) methods. A lived experience advisory group, staff advisory group and project steering committee will support the study throughout. The implementation resources will comprise: advance choice document/advance statement (ACD/AS) documentation, stakeholder trainings, a manual for mental health professionals to facilitate the processes of creating and revising advance statements, and informatics development.

**Discussion:**

The implementation resources will help increase the likelihood of the new mental health legislation in England being implemented effectively; through aligning evidence-based medicine, policy and law to effectively provide positive clinical, social and financial outcomes for Black people, the National Health Service (NHS) and wider society. This study will likely benefit a wider group of people with severe mental illness, as when marginalised groups who are least engaged, can be supported with these strategies, then the strategies are likely to work for others.

## Background

### Advance statements in mental health

Advance Statements/Advance Choice Documents (AS/ACDs) are a type of document that aim to give service users (people that use mental health services) more influence in their decisions over their future care when they are in mental health services [1]. Early formations of ACDs/AS arose in Szasz’s (1982) [2] proposition of a psychiatric will where service users who are involuntarily detained, when well, could document their opposition to receiving coercive treatment if they are to become mentally unwell again or lose capacity [2]. This over time has developed into informal crisis plans or ‘joint crisis plans’ in mental health care, wherein planning for prevention of a mental health crisis can aid in recognising early signs of mental health crises and how to manage them [3–6].

In countries with legislation for advance statements specific to mental healthcare (e.g., USA and Scotland), uptake has been slow and remains low [7]. In England and Wales, the Mental Capacity Act 2005 (MCA) supports the making of ACDs/AS through detailing preferences and wishes for care and advance decisions to refuse treatment (ADRT), that detail determinants of whether a person has capacity to make decisions and the procedures that are entailed when the person lacks capacity [8]. A person lacking capacity refers to their ability to make decisions based on conditions that may affect the function of their brain or mind. When making advance decisions, a person’s consent is needed, and their consent is dictated by their mental capacity [9]. However, the Mental Health Act 1983 (MHA) and the MCA are separate pieces of legislation that address different aspects of the law. The MCA addresses advance decision making however, the MHA focuses on the risk of harm to a person or others and can therefore determine whether an individual receives compulsory treatment. The MHA can override the MCA and therefore, ADRTs and any other ACD/AS can be overridden by the MHA if deemed necessary [9]. Further, there is little evidence that service users have the opportunity to use the advance planning provisions under the MCA [8].

Research in the USA, Scotland and England suggests there to be barriers at service user, clinician and service levels. Completing an ACD/AS involves asserting advance wishes and refusals for preferences for treatment and other aspects of care when one has the capacity to do so [1]. Research in the US and UK shows that the majority of service users need support to do this [8–11]. In Morriss et al.’s (2020) [8] survey of people with bipolar disorder in England, among the third who were aware of the MCA, unrealistic expectations about advance planning and misunderstanding about the different forms (advance treatment refusals, advance statements and power of attorney) were common. Among the 10% who had made an advance refusal, only half were written down, of these, many were not given to anyone else and almost all were reportedly ignored during MHA detention (if detained under the MHA, advance refusals can be overridden if deemed necessary by clinicians, and treatment for mental disorders can be given to a service user without their consent). Scottish evidence suggests some service users are sceptical about whether ACDs/AS will be followed by staff, while others do not acknowledge their relevance [12].

Mental health professionals express reservations about being able to access ACDs/AS and honour the person’s wishes [7, 13, 14]. In the CRIMSON trial of Joint Crisis Plans [3] (JCPs, a type of advance statement), researchers identified three barriers: (1) lack of recognition of the benefits of advance statements; (2) not recognising the need for a change in the clinician-patient relationship including discussing treatment options and supporting patient choice; and (3) difficulties in implementation when working across the healthcare system [3]. Moreover, while some clinicians believed the external JCP facilitator was necessary for empowering service users to participate in shared decision making, others feared interference [15]. However, better quality training on the Mental Capacity Act (MCA) in the UK correlates with greater willingness to discuss Advance Decisions to Refuse Treatment amongst people that have bipolar disorder [16].

Service users, clinicians and carers view ACDs/AS as potentially offering positive outcomes including: reduced coercion or trauma associated with compulsory treatment; building therapeutic alliance [5, 11]; earlier presentation; avoiding harm; enhancing communication; empowering service users and improving clinician confidence [15, 17–19]. Legislation for ACDs/AS is therefore likely to create an implementation gap, and there remains a need for an evidence-based approach to ensure effective implementation.

### Black people in mental health services & relationships with staff

Detention rates of Black people, defined as people of Black African and Caribbean heritage including those of mixed ethnicity, are disproportionately (> 3 times) higher than those of White British people and they have poorer care experiences and outcomes [20, 21]. Black people are more likely to access mental healthcare via the criminal justice system than through primary care [22] or have police involved in their detention [23], with their treatment involving increased use of compulsion, longer admissions and more detention in secure settings [24]. Black people of Caribbean heritage are more likely to be re-admitted or repeatedly detained than White people [20] and are less likely to be referred for specialist mental healthcare [25]. This pattern incurs increased service costs [26] – the current unit cost/day for caring for someone with a psychotic relapse in the community is £146, compared with £455 in hospital. Detaining Black people has been estimated conservatively to cost ~ £158 million/year (Department of Health & Social Care, 2018) [26].

Almost half (47%) of the explanations for variations in care have no or limited supporting evidence [4]. Interventions based on these explanations are likely to fail [4], and current methods of supporting Black people previously detained under the MHA are insufficient (Department of Health & Social Care, 2018) [26]. In contrast, ACDs/AS are the only evidence-based intervention to reduce compulsory psychiatric admission overall, with particular benefit for Black people [27, 28]. They thus represent a way to reduce unwarranted variation by intervening in a negative cycle of dissatisfaction with services [29], impaired therapeutic alliance and trust, disengagement from services, reduced help seeking [25, 26] and repeated compulsory admissions, associated with reduced quality of life.

### Importance of advance statements for Black people

Black people with severe mental illness (SMI) benefit more than other groups from ACDs/AS. The CRIMSON trial showed greater cost-effectiveness of JCPs for Black people compared with White and Asian participants [5], arising from reduced inpatient service use. In a US study [11], completing an ACD/AS was a more empowering experience for African Americans compared with other ethnic groups [30] and demand for these was higher among non-White people [11]. In England, stakeholders found ACDs/AS to be important for Black people, however they may face more barriers in creating them, this includes a lack of trust in mental health services may create a high demand for ACDs/AS among Black people in England [10, 20, 31, 32].

For there to be an improvement in relationships between staff and Black people in mental health services and for Black people to benefit from ACDs/AS, they must be implemented effectively using processes that are informed by: research on the barriers to implementation of ACDs/AS and research focused on and co-produced with Black people and their carers/supporters and mental health professionals.

## Methods

Black service users, carers/supporters and mental health staff/professionals will be invited to participate via means of social media, the National Health Services’ (NHS) mental health service provider’s website, presentations to clinical teams and service providers, adverts, flyers and use of existing partnership agreements with local members of Black communities and organisations.

### Aims

The aim of this study is to co-produce and test an advance statement’s implementation resource for Black people previously detained under the MHA, which can be applied to implement advance statements for all people with SMI in all primary and secondary care mental health services. Specific objectives are as follows, to:

1. Ascertain barriers to and enablers of completing, accessing, honouring and reviewing advance statements by Black service users.
2. Gather stakeholder feedback on pre-existing advance statement templates and modify them.
3. Co-produce guidance and training on advance statement implementation.
4. Co-produce implementation strategies to support the use of the modified advance statement template.
5. Test the implementation of advance statements using the resource developed in Objectives 1–4 against process and service user satisfaction measures.
6. Revise and re-test implementation of advance statements based on results of testing, using Plan-Do-Study-Act Cycles (PDSA)
7. Disseminate the revised implementation resource for advance statements and the study results.

### Design

The study comprises three phases: 1) formative work through stakeholder workshops; 2) co-production of resources and implementation strategies through a Delphi consensus development exercise and working groups; and 3) testing of the resources and implementation strategies using quality improvement (QI) methods.

### Setting

The study sites will be based in four local government areas in South London served by the same National Health Service (NHS) mental health service provider. These sites were selected to build on established ACD/AS resources and because their total populations serve large numbers of Black people (Lambeth 24%, Lewisham 26.8%, Southwark 25.1%, Croydon 22.6% [33].

### Procedures

#### Participants

Eligible participants across all three study phases will be: service users aged 16 + who have been previously detained under the MHA who self-identify as being of Black African or Caribbean heritage, or mixed with Black ethnicity including one of these; informal supporters/carers (18 +) of eligible service users, who are likely to be named in advance statements; professionals likely to be involved in supporting completion and revision of advance statements (community mental health team [CMHT] staff, advocates, peer workers); professionals likely to be involved in referring to advance statements (inpatient and emergency department, home treatment, place of safety and street triage staff); professionals involved in detention under the Mental Health Act (approved mental health professionals (AMHPs) and Sect. 12 approved doctors) who need to access advance statements; and primary care staff including mental health leads, who care for people with SMI discharged from secondary services.

#### Sampling technique and sample sizes

Purposive sampling will be used for all three phases with the aim of recruiting:

- All mental health staff types,
- carers/supporters of any ethnicity, gender and of varying ages and
- Black people of Black African and Caribbean heritage of any gender, varying ages and mental health diagnoses, and to include some with experience of forensic services.

Phase 1 stakeholder workshops will have up to 10 participants per workshop, with a total of 20 service user participants, 30 staff/professionals participants and 10 carers/supporters participants. For the Phase 2 consensus development exercise and coproduction working groups there will be up to 15 participants with a total of 60 participants for Phase 2. For Phase 3 the aim will be to recruit a target sample of 60 participants producing an ACD/AS and those involved in the completion of their advance statement.

#### Recruitment

Recruitment for staff, service users and carers/supporters for all phases of the study will occur through presentation to clinical teams and service providers via presentations to clinical teams and service providers, adverts, flyers to Academic Health Science Network organisations (e.g., the National Health Service (NHS), charity organisations (e.g., Recovery Colleges, Black Thrive), faith-based and community settings (e.g., carer support groups, local churches) and social media platforms.

Service users and carer/supporters will specifically be recruited through: peer support services/groups within the boroughs; flyers distributed physically at service sites and through social media, podcasts and an NHS based app for those using this service provider to potential participants and through the prior listed groups and organisations; the use of existing partnership arrangements with local leaders and members of Black communities and organisations, including voluntary sector groups and faith-based organisations that support Black people’s mental health and wellbeing such as the ON TRAC project – a collaborative project between King’s College London and Black faith community groups in South London – and the Patient and Carers Race Equalities Framework (PCREF) – a strategy that aims to improve the experience of ethnic minority communities experiences of care in mental health services; and the NHS mental health service provider’s intranet; contacting local media such as radios and newspapers; community mental health teams; using the Clinical Records Interactive Search system (CRIS) in collaboration with Maudsley Biomedical Research Centre (BRC) where study information will be sent to patients who have provided Consent for Contact (C4C).

### Procedures

#### Phase 1: Formative work

##### Design

To address Objective 1, six stakeholder workshops will be held for separate groups of staff (2–3 groups), service users (2 groups) and carers/supporters (1 group). Each stakeholder workshop will be lead and facilitated by members of the research team, where participants will be informed of:

- The work of the Independent Review of the Mental Health Act (MHA) in England;
- The government’s acceptance of, and response to, the Review’s recommendation of the introduction of advance choice documents;
- Current projects within the NHS mental health service provider that use advance statements and crisis planning (Crisis Plus and Crisis PACk [10, 17]); evidence for implementation barriers and facilitators.

Questions for the workshops will be developed by the research team and project advisory groups, and will be informed by the teams’ clinical expertise, lived experience of the research team and advisory groups and previous research on the use of ACDs/AS and their use amongst Black service users.

##### Data analysis

The workshops will recorded and transcribed verbatim. The transcripts will be analysed using the framework method [34] to identify common themes from each individual workshop, which will provide provisional recommendations for the implementation resource and issues requiring further discussion during Phase 2. In order to appropriately analyse and understand the current ACD/AS issues all stakeholders experience, so that an informed and systematic approach is taken for Phase 2 in developing the implementation resource [35, 36]. Results of the workshop will be discussed with the Staff Advisory Group and the Lived Experience Advisory Group to inform the design of Phase 2 workshops, and to develop a set of provisional recommendations on the procedures and materials for Phase 3 where the ACD/AS resource will be implemented.

#### Phase 2: Consensus development exercise and coproduction of implementation resource

##### Design

Phase 2 address objectives 2–4 and comprises a consensus development exercise and three co-production workshops for service users, carers and staff meeting the same inclusion criteria as Phase 1 to attend jointly. Participants who contribute to Phase 1 will be asked for their consent to partake in Phase 2.

##### Consensus development exercise

A modified nominal group technique will be used with the results and recommendations from Phase 1 used as the basis for the exercise. This approach will give flexibility for the participants to discuss the complexity of the issues surrounding the recommendations from Phase 1, whilst ensuring critical reflection and the refining of other recommendations [3, 37].

The results from Phase 1 will be presented to expert panellists (target *n* = 12–15) who took part in Phase 1, alongside additionally recruited participants, with the aim of ensuring that 50% of panellists are service users. One round of voting will take place, with facilitated discussion.

##### Analysis

Analysis from the consensus exercise will be carried out and reported, with a descriptive summary of the recommendations that apply to service users, staff/professionals and carers, in addition to site-specific recommendations (strongly supported recommendations =  ≥ 80% Yes and < 20% No votes; fairly supported recommendations =  ≥ 70% Yes and < 20% No votes).

##### Coproduction of implementation resource

The results from the consensus development exercise will be discussed with the two advisory groups. Participants of the stakeholder workshops (Phase 1) and the consensus development exercise, and members of both advisory groups, will then be invited to a series of three workshops to co-produce the implementation resource. The implementation resource will compromise of documentation and trainings for service users, staff/professionals and carers/supporters on completion and use of ACDs/AS.

Between each of the three meetings, the research team will work with the advisory groups, the co-applicants and provider organisation colleagues: a videographer, a graphic designer, trainers, and informatics experts to create: training for mental health staff/professionals; material and a course for service users, carers/supporters and mental health staff/professionals and clinical records access in retrieving and viewing an ACD/AS during crisis. Documents and training materials will be edited following advice from the legal consultant.

##### Data analysis

Data from the consensus development exercise will be recorded, transcribed and analysed by the research team. The transcribed data will be analysed using framework analysis to identify the provisional results and recommendations for the implementation resource; using a framework will ensure that the most dominant recommendations are used for the creation of the implementation resource [36]. The data from the coproduction working groups will not be recorded but notes will be taken by a member of the research team based on the information gained from the working groups, and the feedback will be used to inform the following coproduction meetings and the PDSA Cycles (Phase 3).

#### Phase 3: Implementation – PDSA cycles

##### Design

Phase 3 of the study will use Plan Do Study Act cycles (PDSA), a form of Quality Improvement (QI) methods to test and improve the implementation resource developed in Phase 2 with the aim of learning what further modifications will be needed, addressing objectives 5–7 [35, 38]. The cycles will be conducted monthly over a 6-month period and will comprise a review of data collected: before completing an ACD/AS; after completing an ACD/AS and monthly feedback from staff on the process of helping a service user make an ACD/AS (see Table 1). The training designed through Phases 1 and 2 will be delivered and ACDs/AS will be completed with participating service users using the process and documentation designed.

**Table 1:** Quantitative and qualitative data collection: measures, time points and sources

	**Baseline**	**Post advance statement completion**	**Post event when advance statement use expected**	**Follow up**	**Monthly throughout Phase 3**
Demographic & clinical data	Service users				
Staff role data	Staff involved in completion			Staff involved in completion and/or use	
Satisfaction with and perceived value of advance statements completion		Service users	Service users	Service users Carers/informal supporters involved in completion Staff involved in completion	
Satisfaction with and perceived value of statements use		Service users	Service users	Service users Staff involved in completion and/or event Carers/informal supporters involved in completion	
Reasons for completion and non-completion*	Service users	Service users	Service users	Service users Carers/Informal supporters involved in event / completion Staff involved in event / completion	
Distribution of hard & electronic copies		Staff involved in completion Clinical notes		Staff involved in completion / event	
Accessing advance statement			Service users	Service users Staff involved in completion and/or event Carers/informal supporters involved in completion	
Advance statement choices honoured			Service users	Service users Staff involved in event	
Advance statements choices clearly not honoured			Service users	Service users Staff involved in event	
Reasons for advance statement choices not being honoured			Service users	Staff involved in event	
FIM, IAM & AIM		Service users		Service users Carers involved in completion / event Staff involved in completion / event	
Advance statement content			Electronic record review	Electronic record review	
Use of mental health and emergency services				Service user Electronic record review	
Types of revisions made to advance statement				Electronic record review Service user Staff involved in completion	
Staff feedback from team meetings					Staff involved in completion

##### Data collection

To maximise data collection, access to, honouring and review of ACD/AS there will be a variable follow-up period extending beyond the PDSA cycles, this will be dependent on when each participant is recruited. The follow up data will be collected over 2 months, leaving 2 months for analysis. Table 1 details the measures, time points and the sources for the data collection over the study period.

##### Analysis

Descriptive statistics on implementation outcomes and barriers will be reviewed at monthly staff meetings during the Phase 3 PDSA cycles, together with incidences for individual participants of use of acute services, incidents of violence or self-harm and detention under the Mental Health Act. At the end of the project implementation data will be used to estimate the costs of ACD production and to inform the implementation of the ACD/AS process across the National Health Service (NHS) mental health providers across boroughs in London, UK.

Descriptive statistics on questionnaires, fidelity tools and the rates of completing, accessing, honouring and reviewing AS/ACDs will be collected, with pre and post comparisons of the trust item being undertaken.

#### Project management

A core research team comprising the co-leads and contract research staff will meet weekly to progress study procedures. The whole research team will meet monthly to discuss updates from the core research team and input into study management. The project steering committee will help us engage policy audiences nationally, who will meet every 6 months to advise on the study processes with a view to future scale up and knowledge mobilisation.

#### Patient and public involvement

The staff advisory group will include staff who work across acute and community services in the study setting and will meet quarterly to advise on engagement at all stages of the study to help optimise the participation of staff and service users, this will be chaired by Professor Alan Simpson, a professor in Mental Health Nursing. The lived experience advisory group will comprise Black service users and carers of Black service users, and will be chaired by Steve Gilbert OBE, who has lived experience and will also meet quarterly to advise on recruitment and participation of service users at each study phase.

## Discussion

Data from the PDSA cycles will be used to inform future studies and aid the process of the use, completion and review of ACDs/AS within National Health Service (NHS) mental health providers across boroughs in London, UK.

The implementation resource will likely increase the chance of the new mental health legislation in England, surrounding advance choice documents, being implemented effectively. This through aligning evidence-based medicine, policy and law [4, 5, 7, 8, 10, 12, 27, 28] to provide positive clinical, social and financial outcomes for Black people, the NHS and wider society. Strategies that aid in supporting successful implementation of ACDs/AS will allow for better access and delivery of mental health services for Black people that use these services. This knowledge will likely benefit a wider group of people that have Serious Mental Illnesses (SMI). As most marginalised groups, who often engage with services less can be supported with these strategies, which can enable these strategies to work for other people [10, 25, 32], as those that are most at need are acknowledged and catered to first to ensure that the strategies do work for them, as they are produced with and for them.

## Data Availability

Not applicable.

## Abbreviations

ACD/AS: Advance Choice Document/Advance Statement
AdStAC: Advance Statements for Black African and Caribbean people
ADRT: Advance Decisions to Refuse Treatment
CMHT: Community Mental Health Team
JCP: Joint Crisis Plan
MCA: Mental Capacity Act
MHA: Mental Health Act
NHS: National Health Service
PDSA: Plan Do Study Act
QI: Quality Improvement
SMI: Serious Mental Illness
